# Transcatheter Approach for Critical Pulmonary Stenosis or Pulmonary Atresia with Intact Ventricular Septum in Young Infants Using the Simmons Catheter

**DOI:** 10.1155/2020/4986815

**Published:** 2020-05-19

**Authors:** Jian Wang, Jing Sun, Jian Shen, Jianping Yang, Ling Yang, Pengjun Zhao, Sun Chen

**Affiliations:** Department of Pediatric Cardiology, Xinhua Hospital Affiliated to Medical School, Shanghai Jiaotong University, 1655 Kongjiang Rd, 200092 Shanghai, China

## Abstract

**Methods and Results:**

We retrospectively reviewed 52 young infants, 41 of whom had CPS and 11 had PA/IVS, in a single center from June 2009 to October 2017. Patients were divided into three groups according to the type of catheter used to enter through the RVOT. The unique structure of the Simmons catheter allowed it to be maneuvered directly into the RVOT within a few minutes. Compared with the other two groups, the Simmons catheter group had a significantly shorter fluoroscopy time entering through the RVOT (*P* < 0.001) and a shorter total X-ray exposure time (*P* < 0.001). Furthermore, compared with the floating catheter group, the success rate of surgery was much higher in the Simmons catheter group (*P* < 0.001).

**Conclusions:**

The Simmons catheter is a safe and effective method to enter through the RVOT in infants with CPS or PA/IVS. Therefore, the Simmons catheter could be an alternative catheter when entering through the RVOT in young infants, especially neonates with low birth weight.

## 1. Introduction

Critical pulmonary stenosis (CPS) and pulmonary atresia with intact ventricular septum (PA/IVS) are rare and complex congenital heart defects (CHDs), accounting for approximately 3% of all CHDs [1]. Right ventricular outflow tract obstruction with various degrees of right ventricular hypoplasia is an important determinant of the management strategy [2].

PA/IVS was first managed with percutaneous balloon pulmonary valvuloplasty (PBPV) in 1990 [3]. This technique is now the predominant treatment for patients with proper anatomy suitable for transcatheter intervention in many medical centers [4, 5]. Interventional therapy for PA/IVS during the neonatal period was reported to have a high success rate, with a mid- and long-term survival rate ranging from 81.0 to 92.5% [6, 7]. However, the conventional approach is to enter into the right ventricular outflow tract (RVOT) in young infants. This technique has certain difficulties and requires a long fluoroscopy time [8], especially in cases with hypoplastic right ventricle.

The Simmons catheter was first applied for angiogram in 1973 and has been widely used in the catheterization of the left vertebral artery, brachiocephalic arteries, and some visceral arteries [9]. In this study, we introduced a novel application of the Simmons catheter (Terumo®, mode: EA15010M) to maneuver directly into the RVOT to perform pulmonary valvuloplasty in young infants with CPS or PA/IVS. We compared the efficacy and safety of the Simmons catheter with those of the conventional approaches using the floating catheter and Judkins right coronary (JR) catheter.

## 2. Methods

### 2.1. Study Subjects

We retrospectively reviewed 52 cases of young infants with CPS or PA/IVS admitted to Xinhua Hospital Affiliated to Shanghai Jiaotong University, School of Medicine, from November 2012 to October 2017. All patients received interventional PBPV therapy. The inclusion criteria were as follows: (1) age under 3 months and (2) diagnosis using echocardiography as PA/IVS or CPS with hypoplastic right ventricle (tricuspid valve gradient; TVG >70 mmHg) [10]. Patients with a tricuspid valve (TV) *Z*-score <−2.5 were excluded because of the requirement of a pulmonary blood flow increase [4]. The study was approved by the ethics committee of Xinhua Hospital. Informed consent was obtained from all patients' parents in the form of agreement for catheterization and the use of data collected before and after the procedure.

Patients were divided into three groups according to the type of catheter used to enter the RVOT, which were the Simmons catheter group (*n* = 25), the floating catheter group (*n* = 11), and the JR catheter group (*n* = 16). Echocardiography was reviewed for right ventricular morphology, TV annulus *Z*-score, degree of tricuspid valve regurgitation (TR), pulmonary valve (PV) annulus *Z*-score, and primary diagnosis. Demographic data on catheterization was collected, including total fluoroscopy time, fluoroscopy time entering into the RVOT, success rate of catheterization, age at first intervention, the incidence of complications during catheterization, and the length of hospital stay. Follow-up work included echocardiography and electrocardiogram examinations at 1, 3, and 6 months and 1, 2, and 3 years after catheterization, respectively. Regarding the patients who did not return for the postoperative examination, we telephoned them to collect information on reintervention or major complications. Therefore, we included the data of reintervention as the major indication for long-term follow-up.

### 2.2. Statistical Analysis

Statistical analysis was performed with SPSS 19.0 (IBM Corp., Armonk, NY, USA). A chi-square test, mono factor analysis of variance, or Kruskal-Wallis H-test was applied. A *P* value <0.05 was considered statistically significant.

### 2.3. Catheterization Procedures

Catheterization for PBPV was performed in all patients under general anesthesia. To cannulate the femoral artery and vein, 4F and 5F sheaths were used, respectively. Heparin (100 U/kg) was administered immediately after cannulation. All 11 cases of PA/IVS were membranous atresia, and coronary angiography was performed to exclude right ventricle dependent coronary circulation. All procedures were carried out by a single chief physician in our center with several fixed assistants.

In the floating catheter group, a 5-French floating catheter was advanced through the femoral vein to reach the pulmonary valve via clockwise rotation. In the JR catheter group, a 3-French catheter was used to enter through the RVOT.

**Figure 1:**
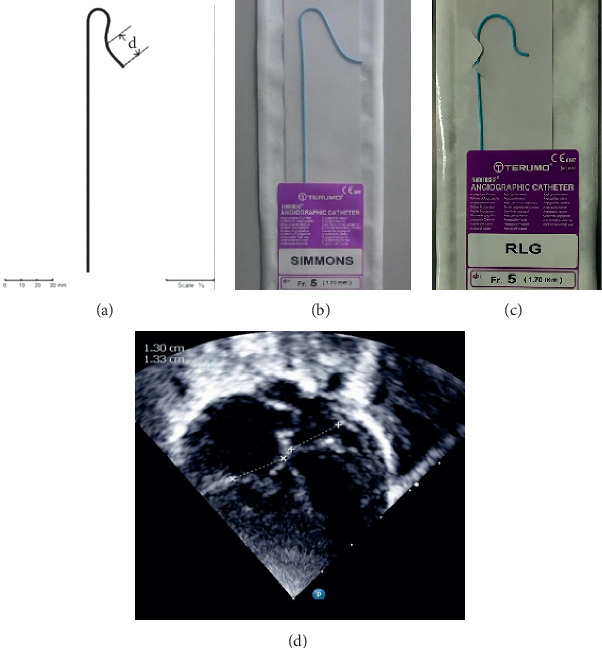
(a) The morphological structure of the Simmons catheter and its distal part. A close-up view of the (b) Simmons catheter and (c) RLG catheter. (d) The measurement of the TV.

**Figure 2:**
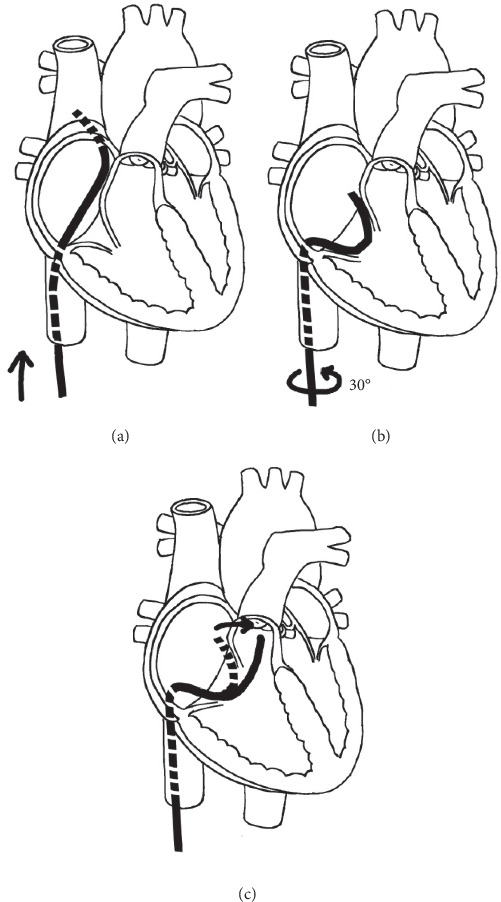
(a) The Simmons catheter is advanced to the right atrium directly. (b) The catheter is rotated counterclockwise about 30°. (c) When the distal part of the Simmons catheter points to the left, it will flip into the RVOT.

**Figure 3:**
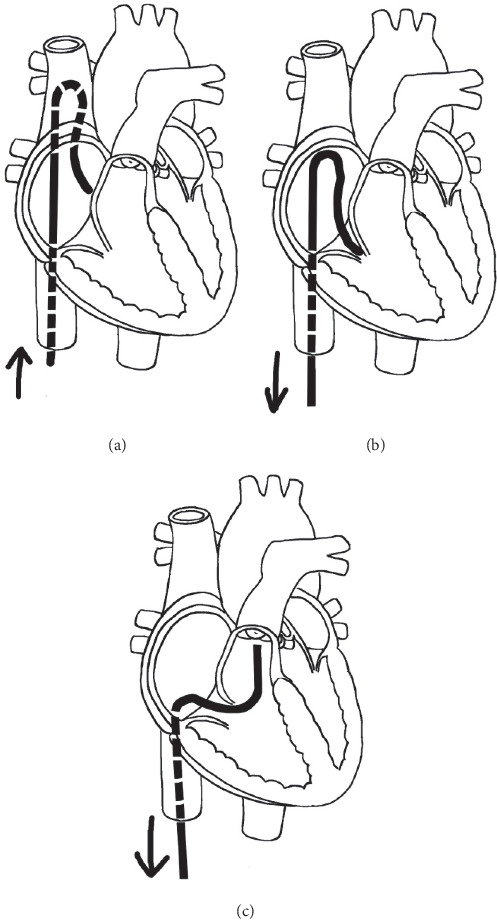
(a) The Simmons catheter is advanced directly into the superior vena cava with its original appearance. (b) The catheter is withdrawn slowly and sent over the TV with an adjusted angle. (c) The catheter will flip into the RVOT automatically.

**Figure 4:**
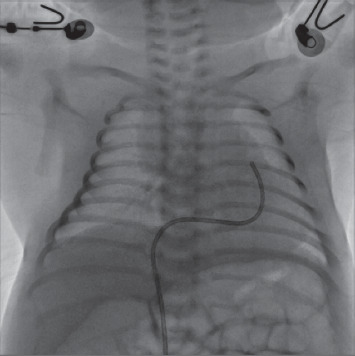
The Simmons catheter was maneuvered directly into the RVOT (anteroposterior view).

In the Simmons catheter group, we used a Simmons catheter to maneuver into the RVOT. We chose a Simmons catheter for catheterization when the distal part of the catheter (Figure 1(d)) was shorter than the diameter of the TV. When the TV diameter was shorter or the right ventricle was more severely hypoplastic, a Rosch Left Gastric (RLG) catheter (Terumo, Japan, Figure 1(c)), which was similar in shape but smaller in size, was chosen. Since these catheters are similar, the results of both the Simmons and RLG catheters were calculated as one group. We used two operative approaches: the first approach was to advance the Simmons catheter directly into the right atrium and rotate the catheter counterclockwise by about 30°. When the catheter tip pointed to the left, the Simmons catheter was sent into the RVOT (Figure 2); the second approach was to advance the Simmons catheter directly into the superior vena cava with its original appearance and then withdraw the catheter slowly until the catheter flipped into the RVOT automatically (Figure 3). The distal end of the Simmons catheter was located in the RVOT with its tip pointing towards the pulmonary valve after the procedure (Figure 4). RVOT angiography was then performed.

**Figure 5:**
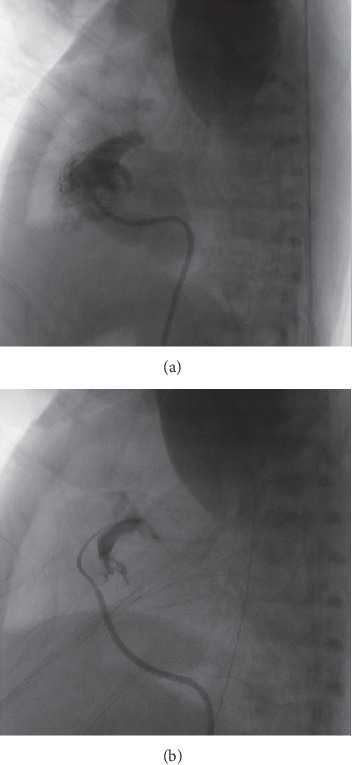
(a) Angiography of the atretic pulmonary valve with the Simmons catheter (lateral view). (b) Angiography with a microcatheter to confirm its position at the center of the pulmonary valve.

**Figure 6:**
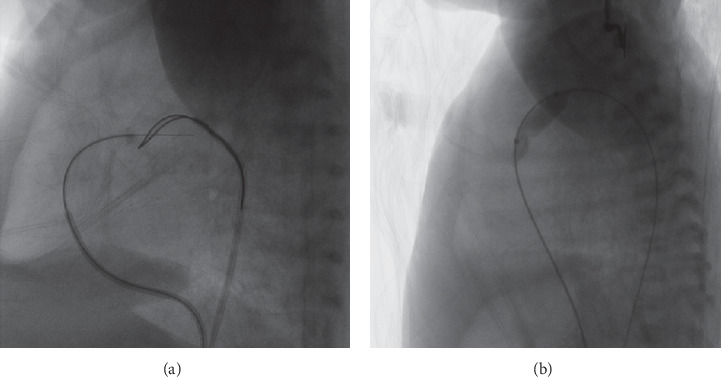
(a). Perforation was performed with the Conquest Pro, and the femoral vein-ductus-femoral artery pathway was built with a snare to trap the microguide wire; (b) balloon dilation was manipulated with a microballoon of a diameter 1–1.2 times that of the pulmonary valve annulus.

With the Simmons catheter, we found it easy to insert a 1.8-F microcatheter and maneuver the distal end of the microcatheter close to the center and perpendicular to the pulmonary valve (Figure 5). Because of the disallowed use of radiofrequency in China, a Conquest Pro 8–20 wire with a 0.2 mm tip was used to perforate the atretic pulmonary valve in patients with PA/IVS. Most of the time, the Conquest Pro 8–20 was replaced with a 0.46 mm microguide wire to establish a femoral vein-ductus-descending aorta-femoral artery pathway. The balloon dilation was manipulated by a microballoon with a diameter 1–1.2 times the pulmonary valve annulus diameter (Figure 6). Finally, the right ventricle pressure was measured, and the right ventricle angiography was performed.

## 3. Results

Patients were divided into three groups: the Simmons catheter group (including 8 cases with the RLG catheter) (*n* = 25, with 21 cases of CPS and 4 cases of PA/IVS), the floating catheter group (*n* = 11, with 8 cases of CPS and 3 cases of PA/IVS), and JR catheter group (*n* = 16, with 12 cases of CPS and 4 cases of PA/IVS). The basic characteristics of patients in the three groups are presented in Table 1. No significant differences were found between the characteristics of the three groups. The TV Z-score was -0.85 ± 0.6 in the floating catheter group, −0.93 ± 0.7 in the JR catheter group, and −0.90 ± 0.7 in the Simmons catheter group. The PV Z-score was −1.97 ± 0.7 in the floating catheter group, −1.87 ± 0.7 in the JR catheter group, and −1.94 ± 1.1 in the Simmons catheter group. Although no significant difference was found, patients in the Simmons catheter group were younger (mean age of 32.5 ± 21.7 days) and had a lower weight (3.5 ± 0.7 kg), including 4 cases of premature infants, with the lowest weight at the time of surgery being 1890 g, compared to the other groups.

All 52 infants with the diagnosis of CPS or PA/IVS received the transcatheter PBPV therapy. The utility of the Simmons catheter made it easier and quicker to advance directly into the RVOT, especially in young infants with low birth weight. The outcomes of PBPV in the three groups are presented in Table 2. According to the Kruskal-Wallis H-test, the fluoroscopy time entering the RVOT in the Simmons catheter group was significantly lower than in the other two groups (*P* < 0.001). In addition, the total fluoroscopy time of the entire catheterization procedure was significantly lower in the Simmons catheter group than in the other groups (*P* < 0.001). The success rate of catheterization was significantly higher in the Simmons and JR catheter groups than in the floating catheter group (*P* < 0.001).

Complications during the procedure were mainly arrhythmia, including ventricular premature (*n* = 3), frequent onset of ventricular tachycardia (*n* = 2), atrial premature (*n* = 2), nonsustained ventricular tachycardia (*n* = 1), and second-degree atrioventricular block (*n* = 1). These complications were more often observed in cases of long procedures when advancing into the RVOT with constant stimulation of the myocardial tissue. There was a trend towards lower complication rates in the Simmons catheter group (*n* = 2, 8.0%) than in the floating catheter group (*n* = 4, 36.3%) and JR catheter groups (*n* = 3, 18.8%), but it did not achieve statistical significance (*P*=0.120). The floating catheter group had seven cases of failed procedure; one failure of PA/IVS was due to an extremely low birth weight and inability to perforate the pulmonary valve, and two failures of CPS were due to the long procedure of advancing into RVOT and the frequent onset of ventricular tachycardia during that period. The other four cases of CPS also failed due to the inability to maneuver the floating catheter into the RVOT after a long period of attempts.

## 4. Discussion

CPS and PA/IVS are rare CHDs; however, they are also the most common types of cyanotic CHD during the neonatal period [11]. The variability in malformation necessitates individualized therapeutic pathways for both CPS and PA/IVS; the therapeutic pathway decided is in accordance with the morphology of the right ventricle and associated abnormalities [12]. An interventional approach is essential in most cases as a first stage procedure to stimulate the growth of the right ventricle and lay the foundation for biventricular correction [13].

In the process of interventional therapy for CPS and PA/IVS, one of the challenges is to stably position the catheter into the RVOT with a small and hypertrophic right ventricle. This usually requires a long fluoroscopy time and complex procedures. At our clinical center, we have previously applied the conventional floating catheter in patients with CPS and PA/IVS to reach the RVOT. The acute angle between the RVOT and the infundibular tract, especially in young infants with a hypertrophic or even hypoplastic right ventricle, resulted in many hours spent trying to reach the RVOT. At other clinical centers, the JR catheter is also widely used to maneuver to the atretic pulmonary valve [5, 14]. However, in our experience, using a JR catheter in young infants also required a long fluoroscopy time, and the stiffness of the distal part of the catheter could lead to tissue injury, which was in accordance with previous studies [8].

In our study, we introduced a novel application of the Simmons catheter to reach the RVOT in patients with CPS or PA/IVS, especially young infants with a hypoplastic right ventricle. We choose a Simmons catheter for catheterization when the diameter of the TV was over 1.3 times that of the distal part of the catheter (Figure 1). When the TV diameter was smaller, we chose the Rosch Left Gastric catheter, which is similar in appearance to the Simmons catheter but has a smaller profile.

Using the Simmons catheter to advance into the RVOT in patients with CPS and PA/IVS has several advantages, especially in young infants with low birth weight. The advantages were mainly embodied in its short procedure time, low incidence of complications during catheterization, and user-friendliness. The average fluoroscopy time of entering into the RVOT was significantly reduced, from around 10–20 mins in the floating and JR catheter groups to only 30 s in the Simmons catheter group (1300.7 ± 679.9 s vs. 130.3 ± 81.0 s vs. 46.1 ± 19.9 s, *P* < 0.001). Furthermore, the total fluoroscopy time dropped significantly in the Simmons catheter group from 30 mins to approximately 15 mins (38.2 ± 14.1 mins vs. 19.9 ± 10.5 mins vs. 14.5 ± 4.0 mins, *P* < 0.001). Although not statistically significant, there was a trend towards lower complication rates in the Simmons catheter group than in the other groups (36.3% vs. 18.8% vs. 8.0%, *P*=0.120). In most cases, the complications of arrhythmia during the procedure were caused by constant stimulation of the RVOT. The application of the Simmons catheter greatly minimized the probability of stimulation for arrhythmia by significantly reducing the procedure time to enter into the RVOT. In addition, Simmons catheters are user-friendly during surgeries, with two approaches described in the Methods section above, providing more options for surgeons.

### 4.1. Limitations

Our study has several limitations. First, this was a single-center study with limited sample size. Second, patients were not randomly assigned to each group, causing a potential for selection bias. Third, the procedures were performed by one chief physician; thereby, the accumulation of experience might affect the procedural time and outcome in all groups. Further clinical trials are required to test these results at different centers.

## 5. Conclusions

In this study, we introduced a novel application of the Simmons catheter in patients with CPS and PA/IVS, especially young infants with low birth weight and hypoplastic right ventricle. This catheter structure permitted a rapid approach to the RVOT and significantly reduced fluoroscopy time and thus increased the success rate of the intervention.

## Figures and Tables

**Table 1:** Basic patient characteristics in the Simmons catheter, floating catheter, and JR catheter groups.

	Floating catheter (*n* = 11)	JR catheter (*n* = 16)	Simmons catheter^#^	*P* value
Gender:				
Male	6	8	14	0.934
Female	5	8	11	
Age (days)	43.6 ± 28.6	31.2 ± 20.8	32.5 ± 21.7	0.289
Diagnosis:				
PA/IVS	3	3	4	0.658
CPS	8	13	21	
Gestational age (weeks)	38.4 ± 2.7	38.0 ± 2.6	37.8 ± 2.8	0.538
Weight at initial procedure (kg)	3.7 ± 0.6	3.7 ± 0.6	3.5 ± 0.7	0.511
TV Z-score	−0.85 ± 0.6	−0.92 ± 0.7	−0.90 ± 0.7	0.912
PV Z-score	−1.97 ± 0.7	−1.88 ± 0.8	−1.94 ± 1.1	0.897

^*∗*^PA/IVS: pulmonary atresia with intact ventricular septum, CPS: critical pulmonary stenosis, TV: tricuspid valve, PV: pulmonary valve. ^#^Simmons Catheter group: including 8 cases of the RLG catheter.

**Table 2:** Catheterization outcomes of PBPV in the three groups.

	Floating catheter (*n* = 11)	JR catheter (*n* = 16)>	Simmons catheter^#^ (*n* = 25)	*P* Value
Fluoroscopy time of entering RVOT (s)	1300.7 ± 679.9	130.3 ± 81.0	46.1 ± 19.9	<0.001
Total fluoroscopy time (min)	38.2 ± 14.1	19.9 ± 10.5	14.5 ± 4.0	<0.001
HLOS (days)	12.0 ± 8.9	11.0 ± 5.1	12.4 ± 11.4	0.693
Complications (%)	4(36.3)	3(18.8)	2(8.0)	0.120
Success rate of catheterization				
Success/All (%)				
Reintervention rate (%)	4(36.3)	3(18.8)	6(24.0)	0.582

^*∗*^PBPV: percutaneous balloon pulmonary valvuloplasty, RVOT: right ventricular outflow tract, HLOS: hospital length of stay. ^#^Simmons Catheter group: including 8 cases of the RLG catheter.

## Data Availability

All data generated or analyzed during this study are included in this article.
